# C10ORF99 (GPR15L) increases susceptibility to colitis and colitis-induced colorectal cancer via GPR15-independent mechanisms, while mediating GPR15-dependent T cell migration to the large intestine

**DOI:** 10.1016/j.mucimm.2025.08.001

**Published:** 2025-08-13

**Authors:** Gerald J. O’Connor, Rachel M. Wigmore, Nguyen T. Van, Jihae C. Choi, Karen Zhang, Charles Kivolowitz, Alessandra Chen, Manju Ambelil, Dan R. Littman, Sangwon V. Kim

**Affiliations:** a Department of Microbiology and Immunology, Sidney Kimmel Medical College, Thomas Jefferson University, Philadelphia, PA, USA; b Sidney Kimmel Comprehensive Cancer Center, Jefferson Health, Philadelphia, PA, USA; c US Digestive Health, Exton, PA, USA; d Department of Cell Biology, New York University School of Medicine, New York, NY, USA; e Howard Hughes Medical Institute, New York, NY, USA

**Keywords:** C10ORF99, GPR15L, GPR15LG, GPR15, Migration, Large intestine, Epithelium, Colitis, Colorectal cancer

## Abstract

GPR15 is a homing receptor important for T cell migration to the large intestine, the primary site of inflammation in ulcerative colitis. Both GPR15 and its ligand, C10ORF99, represent potential therapeutic targets for the treatment of IBD; however, the roles of C10ORF99 in the large intestine are not fully elucidated. Here, we demonstrate that C10ORF99 is the non-redundant ligand of GPR15 mediating T cell migration to the large intestine. Furthermore, we demonstrate that C10ORF99 has GPR15-independent functions in the large intestine: C10ORF99 deficiency is protective in chemically induced colitis, and this appears to result from enhanced epithelial barrier regeneration. We found that C10ORF99 can inhibit intestinal epithelial proliferation in a cell-intrinsic manner. Additionally, due to this protection from colitis development in the absence of C10ORF99, C10ORF99 KO is also protected from colitis-associated colorectal cancer development. These data indicate that the deficiency of C10ORF99 can not only block pathogenic T cell migration to the large intestine, but can also promote epithelial barrier repair, potentially offering additional advantages for recovery from ulcerative colitis.

## Introduction

The intestinal epithelium is a single layer of cells separating the microbes in the lumen from host tissues. Disruptions to this layer can result in inflammatory responses culminating in inflammatory bowel disease (IBD).^1^ There are two types of IBD, Crohn’s disease (CD) and ulcerative colitis (UC). CD can impact both the small and large intestines, with areas of healthy tissue between inflamed regions, and it causes inflammation in all layers of the bowel wall. In contrast, UC only affects the large intestine and causes superficial damage to the mucosal layer.^1,2^ The occurrence of IBD appears to necessitate an interplay of genetic, environmental, dietary, and microbial factors.^2–4^ This disease not only diminishes quality of life, but can also result in serious complications potentially requiring surgical bowel resection, extra-intestinal symptoms,^5^ or even the development of colorectal cancer (CRC).^3,6,7^ In 2017, there were an estimated 6.8 million people with inflammatory bowel disease globally, representing a substantial increase in cases from 3.7 million in 1990.^8^ Yet despite the introduction of new treatments such as anti-tumor necrosis factor antibodies, many patients are still refractory to interventions.^3^ This indicates the need for new therapeutic targets.

Compared to the small intestine, the large intestine has significantly more microbiota as well as T regulatory cells (Tregs).^9–12^ Additionally, the small and large intestines have separate mechanisms for lymphocyte trafficking. In the small intestine, lymphocyte migration is regulated through the chemokine receptor, CCR9^13,14^ and its ligand, CCL25. However, T cell trafficking to the large intestine is regulated by the homing receptor GPR15.^15^ This makes GPR15 an important potential target for future therapies to prevent UC, where the large intestine is a primary location of inflammation. GPR15 is a class A GPCR initially investigated as a co-receptor for HIV.^16^ However, its role in maintaining large intestine homeostasis was discovered when it was identified as a colon-homing receptor.^15^ It is largely responsible for trafficking T cells to the large intestine and can direct both Tregs as well as T effector cells (Teff).^15,17^ GPR15-expressing Tregs were shown to be essential in rescuing mice from *Citrobacter rodentium*-induced colitis.^15^ The number of GPR15-expressing T cells could also be increased via the AhR pathway by administering a tryptophan-high diet.^18^ A ligand for this homing receptor is the chemokine-like protein C10ORF99 (also known as GPR15L, GPR15LG, or AP-57).^16,19,20^

C10ORF99 is a small, secreted peptide and is expressed highly at steady state in the large intestine, the oral cavity, esophagus, cervix, and testes.^20^ It is also expressed temporarily in the skin during the development or inflammation; however, it is not expressed in the small intestine.^20,21^ While the *in vivo* function of C10ORF99 has not been demonstrated in the large intestine, it has been shown to mediate T cell migration during the development and the inflammation in the skin.^19,20^ C10ORF99 has also been suggested to affect the proliferation of epithelial cells in a GPR15-independent manner, but with conflicting roles: anti-proliferative functions,^22^ and pro-proliferative functions.^21,23–25^ Since C10ORF99 is one of the most upregulated genes in psoriatic lesions,^21^ most of these proposed roles of C10ORF99 unrelated to homing were studied in the skin. However, these studies were performed in the presence of GPR15, making it difficult to exclude the impact of GPR15-mediated homing effects on the observed phenotype of C10ORF99-deficient mice. Therefore, to confirm the validity of the GPR15-C10ORF99 pathway as a therapeutic target for UC, it is necessary to determine whether C10ORF99 mediates T cell migration to the large intestine via GPR15 and whether it has an additional GPR15-independent function in the large intestine, as suggested in the skin.

Here, we have identified C10ORF99 functions as a non-redundant ligand of GPR15, mediating T cell migration to the large intestine. In addition, we have discovered an *in vivo*, GPR15-independent function of C10ORF99 that reduces the regeneration rate of intestinal epithelial cells in the large intestine. Consequently, C10ORF99 increases the susceptibility of mice to dextran sodium sulfate (DSS)-induced colitis. C10ORF99 can inhibit intestinal epithelial proliferation in a cell-intrinsic manner in vitro, while the impacts of microbial, immune, or other environmental factors remain to be determined. Due to its role in increasing susceptibility to colitis, C10ORF99 also increases the development of colitis-associated colon cancer. Overall, our findings provide novel insight into the understudied large intestine-specific mucosal barrier regulations and a greater understanding of the large intestine-specific lymphocyte homing.

## Results

### C10ORF99 is the non-redundant ligand of GPR15 in the large intestine, mediating T cell migration

**Fig. 1.**
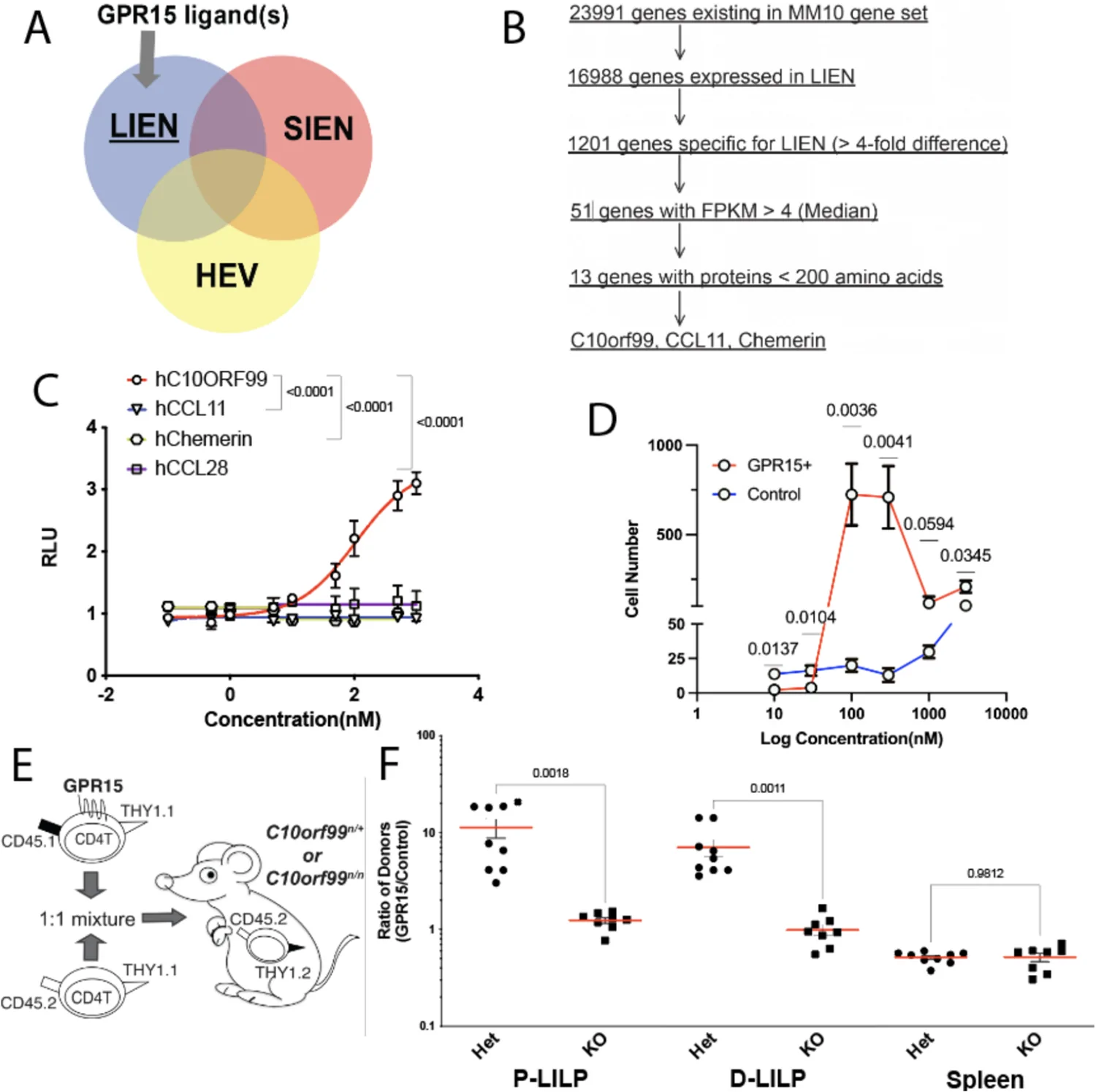
C10ORF99 is the non-redundant ligand of GPR15 for the large intestine. **(A and B)** Schema of the strategy used to identify the ligand of GPR15 from bulk RNAseq data, as well as resulting genes that fell into the established criteria. **(C)** β-arrestin reporter assay results showing signal responses to various chemokines. **(D)** Transwell assay results observing the ability of GPR15-expressing WeHi cells to move to a separate compartment with C10ORF99, compared to control cells. **(E)** Schema of a T cell transfer of GPR15-expressing cells compared to controls into cohoused *C10orf99* Het vs. *C10orf99* KO mice. **(F)** Results of a T cell adoptive transfer showing GPR15-expressing cells compared to controls in the Proximal Colon (P-LILP), Distal Colon (D-LILP), and Spleen (n = 8–9 with littermate controls). Data are shown as mean ± SEM. Populations were compared using Student’s *t*-test.

GPR15 was de-orphaned through the discovery that C10ORF99 can bind to GPR15.^19,20^ We have also confirmed this independently through RNA sequencing. Considering that integrins and GPCRs cooperate to facilitate the exit of lymphocytes from the blood vessels to the peripheral tissues and that integrin α4 and GPR15 work together to mediate T cell homing to the intestine,^15^ we reasoned that the ligand of GPR15 is likely to be selectively expressed in the large intestine blood endothelial cells, but not in the ones in the small intestine. Therefore, we isolated endothelial cells from large intestine, small intestine, and high endothelial venules (HEV) in the lymph nodes, and performed bulk RNAseq (Fig. 1 A and S1A). The criteria for potential homing ligands for GPR15 included genes that were expressed specifically in the large intestine endothelial cells (LIEN) and not found in the small intestine endothelial cells (SIEN) or HEVs, which would be indicated by a 4-fold difference in expression as seen during RNA sequencing. Additionally, these genes would need to code for a protein less than 200 amino acids since GPCRs close to GPR15 phylogenetically have short protein ligands.^26^ We found that *C10orf99, CCL11*, and *Chemerin* fit the criteria (Fig. 1B). Among them, *C10orf99* was the only one with expression below the detectable level in the small intestine endothelium, indicating that it may have a large intestine-specific function. Human GPR15 was activated only by human C10ORF99 during a β-arrestin reporter assay (Fig. 1C), and a Transwell experiment demonstrated that GPR15-expressing cells would move to a compartment with C10ORF99 more than control cells (Fig. 1D). This confirmed that C10ORF99 can function as a ligand of GPR15, consistent with two previous studies.^19,20^ However, while the *in vivo* function of C10ORF99 for mediating T cell homing has been demonstrated in the skin during the development and inflammation,^19,20^ it has not been investigated whether C10ORF99 facilitates GPR15-mediated T cell homing to the large intestine, where it is constitutively expressed. To test this, we have generated *C10orf99* knockout (KO) mice (Fig. S1B) and examined the T cell frequency in the large intestine at steady state. We found that GPR15-GFP^+^ T cells are significantly reduced in *C10orf99* KO mice (Fig. S1C) similar to the reduction in *GPR15*^*gfp/gfp*^ mice.^15^ In addition, we conducted a competitive T cell homing assay *in vivo* by transferring T cells expressing GPR15 as well as control T cells to either *C10orf99* heterozygous (*C10orf99* Het) or *C10orf99* KO mice (Fig. 1E). We observed that GPR15-expressing cells moved to the large intestine 10 times more than control cells (Fig. 1F). However, this was only observed when transferred into *C10orf99* Het mice, but not in *C10orf99* KO mice, indicating that C10ORF99 is a non-redundant ligand for GPR15 as a homing mediator for the large intestine. We also noted that this was true both for the proximal and distal colon, indicating that there are no regional differences for C10ORF99 in mediating GPR15-dependent homing (Fig. 1F). Together, these data newly demonstrate that C10ORF99 non-redundantly mediates GPR15-dependent migration of T cells to the large intestine *in vivo*. C10ORF99 expression in the endothelium of the large intestine likely mediates this function by helping GPR15^+^ T cells exit the blood vessels.

### C10ORF99 deficiency is protective during chemically induced colitis

**Fig. 2.**
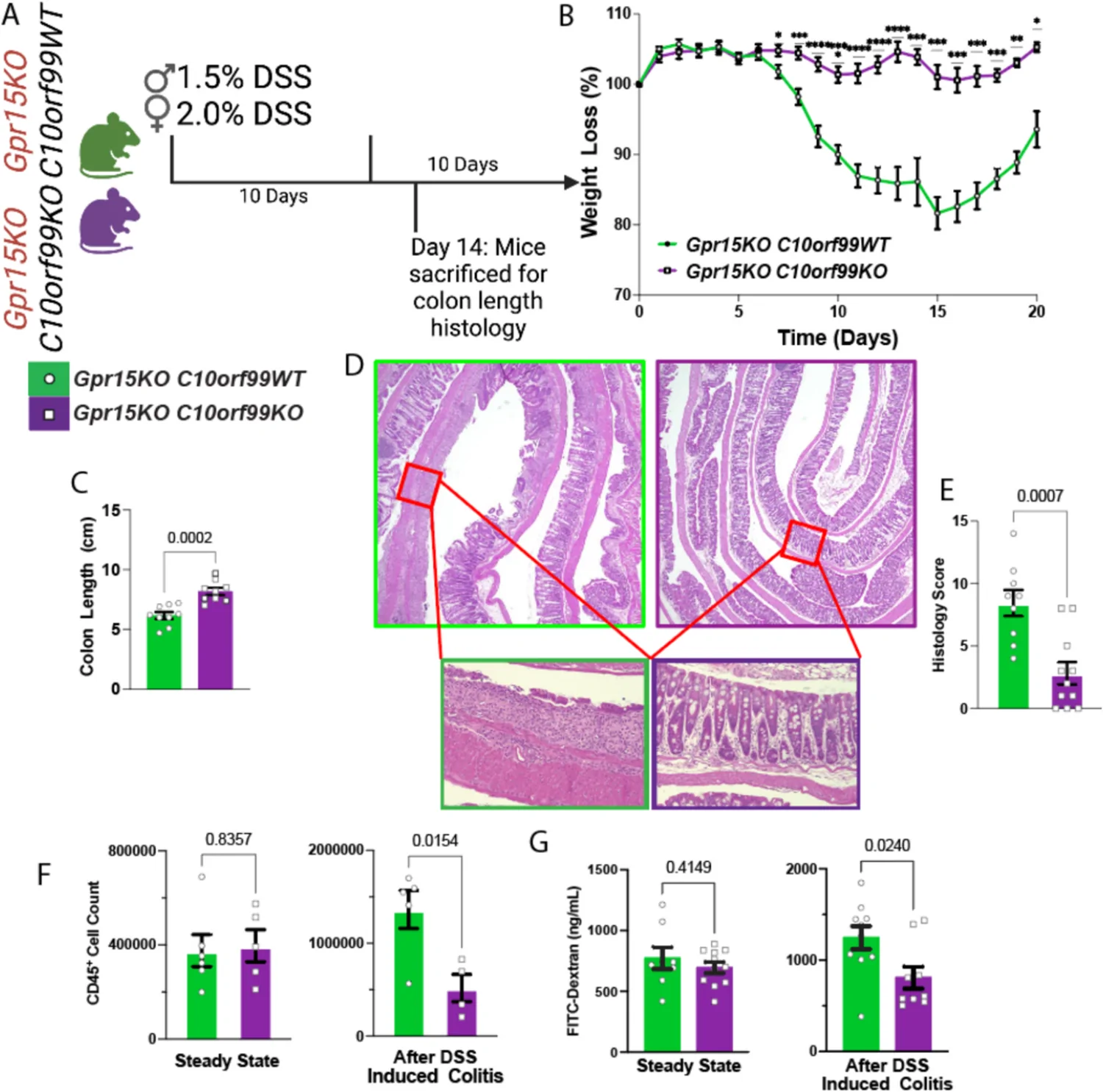
C10ORF99 deficiency is protective during chemically induced colitis. **(A)** Schema of experimental procedure supplying 1.5 % DSS or 2 % DSS to cohoused SKO and DKO mice for 10 days. **(B)** Weight loss of mice following administration of DSS was recorded as a percent of the original weight (n = 20). **(C)** Average colon length from the end of the cecum to the rectum (n = 9). **(D)** Representative images and **(E)** histology score of colon sections following DSS (Histology score criteria found in Table 1) (n = 9). **(F)** Flow cytometry data of infiltration of CD45^+^ cells into colon tissue, looking both at steady state (left) (n = 5) and following DSS (right) (n = 4–5). **(G)** Permeability of the colon at steady state (left) (n = 7,10) and after DSS (right) (n = 9) as measured by FITC-Dextran concentration in serum following a 16 mg gavage. Each dot represents an individual mouse in C, E, F, G. Data are shown as mean ± SEM. Populations were compared using Student’s *t*-test. Cohoused mice were used for these experiments.

Considering the effective therapeutic usage of Vedolizumab blocking the actions of integrin α4β7 in mediating T cell migration to the large intestine in UC,^3,27^ the GPR15-C10ORF99 pathway may be an alternative therapeutic target for UC. However, C10ORF99 is highly induced in the psoriatic lesions, and many studies have proposed that C10ORF99 may exhibit GPR15-independent functions in the skin. Therefore, it is important to determine whether C10ORF99 also plays such a role in the large intestine, the major site of its expression at steady state. However, the problem in investigating the GPR15-independent function of C10ORF99 *in vivo* has been the unavailability of tools to study the function of C10ORF99 without GPR15, such as a *Gpr15, C10orf99 double knockout* mouse (DKO). Thus, to determine whether the GPR15-independent effects of C10ORF99 on the large intestine exist, we created these DKO mice and evaluated their susceptibility to DSS-induced colitis in comparison to our *Gpr15* knockout*, C10orf99* wild-type (SKO) mice (Fig. 2A). The SKO mice exhibited the expected symptoms of colitis: a significant weight loss (Fig. 2B), a shortened colon length (Fig. 2C), and the destruction of the organized crypt architecture (Fig. 2D and E). However, to our surprise, the DKO mice were nearly completely protected from colitis with no loss of weight (Fig. 2B) and fully intact crypt architecture (Fig. 2D and E). At steady state, we did not observe any difference in the number of immune cells in the large intestine between the SKO mice and DKO mice. In contrast, after DSS treatment, we noted that there were more immune cells in the colon of the SKO mice as compared to the DKO mice, indicating that significant colonic epithelial damage is required to observe increased immune cells in SKO mice (Fig. 2F and S3). As these mice were on a *Gpr15* KO background, these effects could not be attributed to alterations in GPR15-dependent immune cell trafficking and thus indicate previously unappreciated GPR15-independent functions of C10ORF99 in the large intestine. To ensure that this phenotype was not an artifact of GPR15 deficiency, we also evaluated the DSS colitis susceptibility between *C10orf99* WT and *C10orf99* KO mice in the presence of GPR15. We found that the *C10orf99* KO mice still exhibited resistance to weight loss from colitis, in contrast to *C10orf99* WT mice (Fig. S2A). The reduction in effect size seen in *Gpr15* WT mice after DSS treatment is likely attributable to the presence of GPR15^+^ T cells in the large intestine, predominantly consisting of T regulatory cells.^15^ To determine whether this difference in colitis susceptibility still exists when colitis is initiated by immune responses rather than epithelial damage, we utilized trinitrobenzene sulfonic acid (TNBS)-induced colitis. In this case, we found that the DKO mice exhibited no protective effects, in contrast to DSS-induced colitis (Fig. S2B). These data indicate that the difference in colitis susceptibility in the absence of C10ORF99 is potentially attributable to alterations in responses to epithelial cell damage, rather than changes in the immune responses within the colon. This was supported by flow cytometry data of various cell populations showing minimal differences between the two groups of mice at steady state (Fig. S3B). While we observed fewer immune cell numbers in DKO mice compared to SKO mice after DSS treatment (Fig. S3C), considering no difference in TNBS-induced colitis (Fig. S2B) and immune cell numbers at steady state (Fig. S3B), we reasoned that this is more likely a consequence of the less epithelial damage that occurred in DKO mice rather than the cause of their colitis resistance. We next examined whether C10ORF99 could influence the permeability of the intestinal epithelium through FITC-Dextran gavage and subsequent serum measurement. We observed no differences in serum FITC-Dextran under steady state conditions (Fig. 2G), indicating that C10ORF99 does not influence epithelial permeability. Rather, we only observed increased permeability in the SKO compared to the DKO (Fig. 2G) following the DSS-induced destruction of typical intestinal architecture (Fig. 2D), indicating that the significant epithelial damage precedes the increased permeability in SKO mice. Altogether, these data reveal a previously unrecognized GPR15-independent effect of C10ORF99 in DSS-induced colitis, wherein a deficiency can confer unexpected protection; however, this is unlikely to be attributable to GPR15-independent immune cell trafficking or alterations in epithelial permeability at steady state.

### C10ORF99-deficient mice have increased proliferation in intestinal crypts

**Fig. 3.**
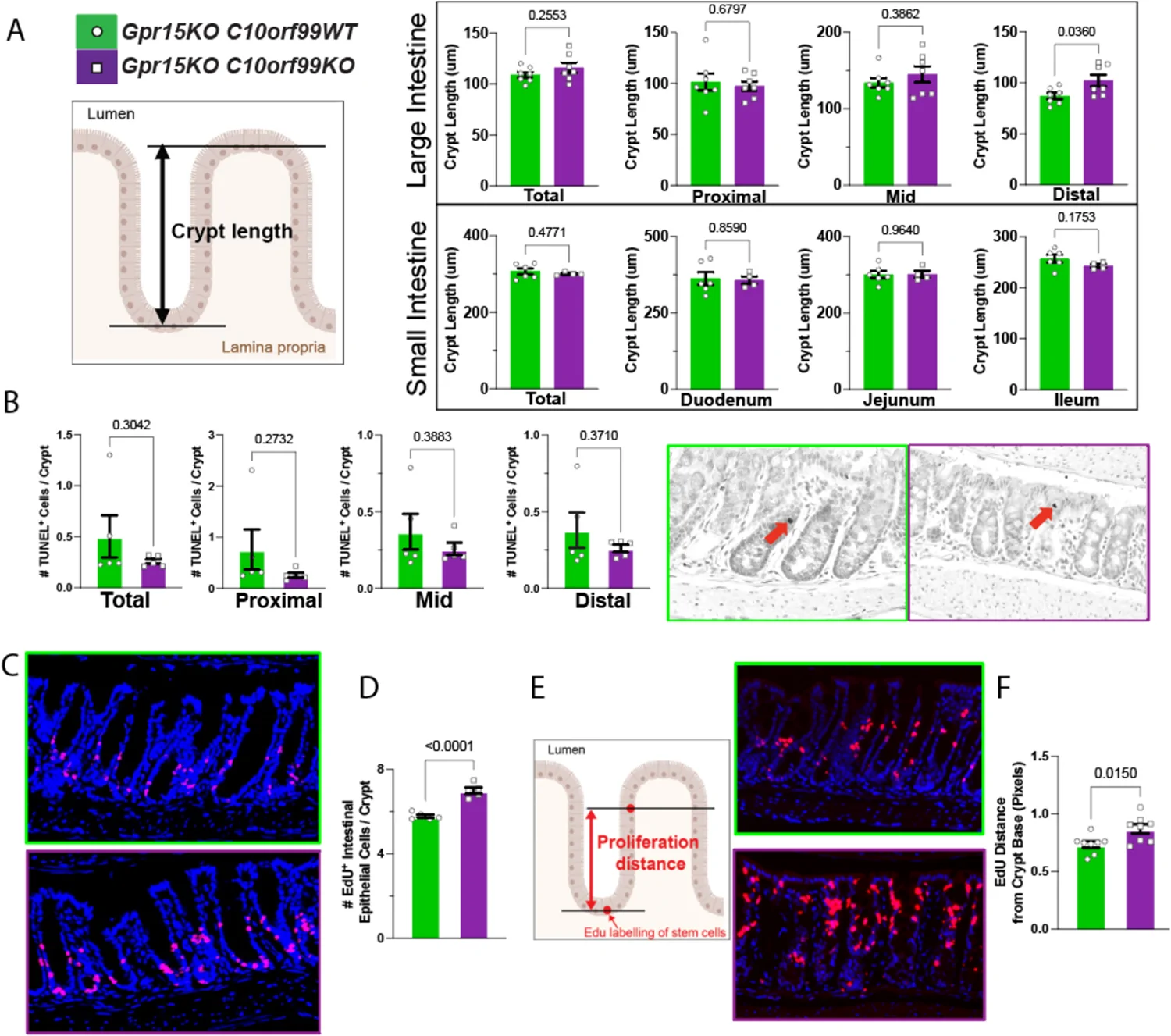
C10ORF99 reduces colonic epithelial proliferation *in vivo*. **(A)** Quantitative analysis of large (top) (n = 7) and small (bottom) (n = 4,6) intestine crypt lengths. **(B)** Measurements and representative pictures of TUNEL^+^ cells per intestinal crypt from colon sections of SKO and DKO mice looking at all colon regions (n = 5). **(C-D)** Representative immunofluorescent staining and counts of EdU^+^ cells within intestinal crypts from colon sections of SKO and DKO mice looking at all colon regions (n = 5). **(E-F)** Representative immunofluorescent staining and distance measurements from the crypt base to the halfway point of EdU^+^ cells from colon sections of SKO and DKO mice looking at all colon regions (n = 8). Data are shown as mean ± SEM. Populations compared using Student’s *t*-test. Each data point shown represents an individual mouse. Cohoused mice were used for these experiments.

Having observed no differences in immune cell trafficking or FITC-Dextran translocation at steady state, or TNBS-induced colitis, we next sought to investigate if there were changes in the process of epithelium regeneration that could explain the differing responses between the SKO and DKO to DSS-induced colitis. We measured the length of intestinal crypts in SKO and DKO mice^28,29^ and found that the DKO mice exhibited an increase in crypt length relative to SKO mice, particularly in the mid and distal colon (Fig. 3A, S2E). This phenotype was not observed in the small intestine, where C10ORF99 is absent (Fig. 3A). The length of intestinal crypts is influenced by the proliferation of epithelial cells at their base and the rate of cell death at their tips^29,30^; thus, crypt length differences indicate changes in either process. We first used TUNEL staining to determine whether the increased crypt length in DKO mice resulted from decreased apoptosis. We found no difference in a number of apoptotic cells per crypt between SKO and DKO mice (Fig. 3B). No changes to apoptotic cells indicated that the increased crypt lengths in DKO mice may be attributed to an enhanced proliferation rate in the absence of C10ORF99. Thus, we administered 5-ethynyl-2′-deoxyuridine (EdU) to SKO and DKO mice and quantified the number of cells per crypt that incorporated EdU. We observed increased EdU^+^ cells in the DKO mice (Fig. 3C and D), indicating an enhanced proliferation rate in the absence of C10ORF99. To further support this, we conducted a pulse chase experiment with EdU, and subsequently measured the distance traversed by the EdU^+^ cells after 48 h.^28,29^ The DKO mice showed a greater migratory distance following injection (Fig. 3E and F), corroborating that epithelial cell proliferation was increased in the absence of C10ORF99. These data suggest that C10ORF99 can inhibit the proliferation of intestinal epithelial cells. Consistent with this, it has been observed that C10ORF99 has the potential to inhibit the proliferation of several cell lines from colorectal cancer *in vitro*.^22^ Thus, we propose that a deficiency of C10ORF99 is protective against DSS-induced colitis because a higher proliferation rate of intestinal epithelial cells allows the faster regeneration of the intestinal epithelium and increases barrier integrity after the epithelial damage upon DSS treatment.

### C10ORF99-mediated inhibition of proliferation does not require microbiome, immune cell, or stromal cell involvement

**Fig. 4.**
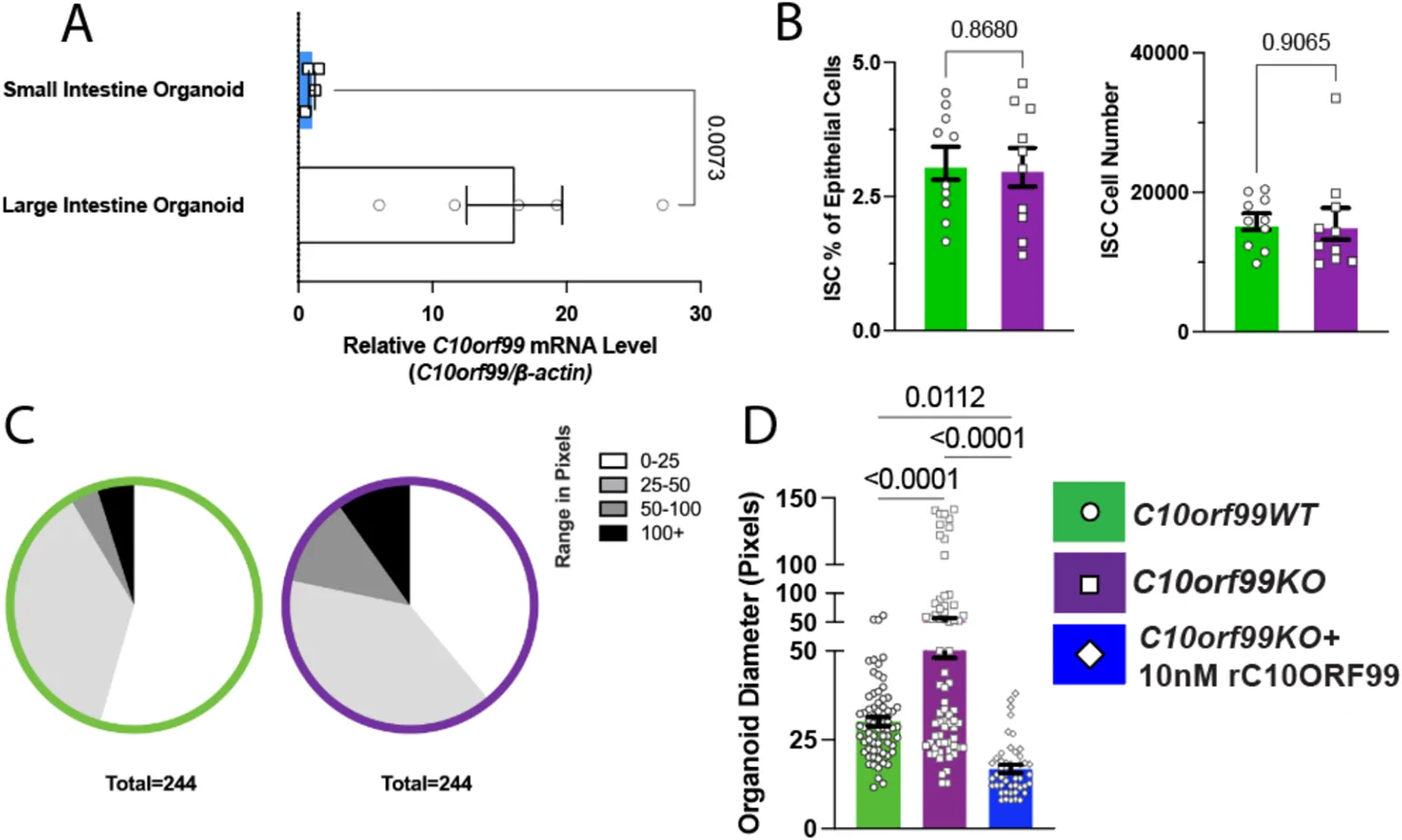
C10ORF99-mediated inhibition of proliferation does not require microbiota, immune cell, or stromal cell involvement. **(A)** RT-PCR comparing *C10orf99* expression between large and small intestine organoids (n = 4–5). **(B)** Flow cytometry data showing LGR5^+^ ISCs as a percentage of total epithelial cells and LGR5^+^ ISC number between *C10orf99* WT and *C10orf99* KO cohoused mice (n = 10). **(C)** Range of organoid sizes between *C10orf99* WT and *C10orf99* KO. **(D)** Organoid diameter between *C10orf99* WT and *C10orf99* KO organoids with or without recombinant C10ORF99 (10 nM) added to the media (n = 70,70,47). Data are shown as mean ± SEM. mRNA levels, ISC percentage, and number were compared with student *t*-test. Organoid diameters were compared with ordinary one-way ANOVA followed by Tukey’s multiple comparisons test. Cohoused mice were used.

Next, we wanted to determine the requirements for the observed phenotype. As the colonic epithelium receives signals from microbiota, immune cells, stromal cells, and other outside influences, we sought to determine if C10ORF99′s inhibition of epithelial cell proliferation was a cell-intrinsic or extrinsic effect. Therefore, we first determine whether C10ORF99 expression in the colonic epithelium is affected by microbiota. For example, because C10ORF99 has been suggested to have antimicrobial properties,^31,32^ it is possible that the *in vivo* phenotype of increased epithelial cell proliferation in the absence of C10ORF99 was caused by C10ORF99-induced alterations to the microbiota. Colonic organoids of epithelial cells grown in vitro without microbiota express *C10orf99*, while small intestine-derived organoids do not (Fig. 4A). This was consistent with previous findings that *C10orf99* expression does not require microbiota.^19^ We next determined if C10ORF99 affects the regeneration rate of intestinal epithelium by altering the number of LGR5^+^ intestinal stem cells (ISC). To accomplish this, we crossed our *C10orf99* KO mice with LGR5-GFP reporter mice^33^ to develop LGR5-GFP reporter mice in the absence of C10ORF99. We found that both *C10orf99* WT and *C10orf99* KO mice had the same number and percentage of LGR5^+^ ISCs (Fig. 4B). Next, to examine the proliferation rate of intestinal stem cells, we sorted for LGR5^+^ ISCs from the colon and developed *C10orf99* WT and *C10orf99* KO organoids. We found that more *C10orf99* KO organoids grew to a greater diameter than those derived from the *C10orf99* WT mice (Fig. 4C and D), indicating that the increased proliferation of intestinal epithelial stem cells in the absence of C10ORF99 is likely a cell-intrinsic phenotype. The addition of recombinant C10ORF99 (rC10ORF99) to *C10orf99* KO organoids reduced the organoid diameter, confirming that the phenotype of *C10orf99* KO organoids can be rescued by the exogenous C10ORF99 (Fig. 4D). We also confirmed our *ex vivo* results through *in vitro* testing of human colon cancer SW480 cells in MTT assays. We found that adding rC10ORF99 to the SW480 cells led to a reduction in conversion to formazan, indicating a potential reduction in cell proliferation (Fig. S2C). This data was also supported by wound healing assays showing reduced wound closure after 4 h when mouse rectal tumor cells (CMT93 cells) were treated with 100 nM mouse rC10ORF99 (Fig. S2D). While this data confirms that C10ORF99 can reduce proliferation without requiring microbiota, it does not prove that microbial shifts cannot influence the effect of C10ORF99 on epithelial proliferation. Thus, to determine the effect of microbiota on C10ORF99-mediated *in vivo* phenotype, we exposed SKO and DKO mice to two weeks of antibiotic-treated water (0.5 g/L Vancomycin, 1 g/L ampicillin, 1 g/L metronidazole, 1 g/L neomycin) and measured intestinal crypt lengths. Upon antibiotic treatment, DKO mice still showed an increase in crypt lengths in the mid colon and a relative reduction in the proximal colon, as in the results when antibiotic treatment was not used (Fig. S2E). However, increased crypt lengths in the distal colon did not appear when antibiotics were administered (Fig. S2E). Overall, this data suggests that C10ORF99 can influence epithelial proliferation without requiring the microbiota’s influence, but significant changes to the microbiota can mask C10ORF99′s influence, particularly in the distal colon.

### C10ORF99 exacerbates colitis-associated colorectal cancer

**Fig. 5.**
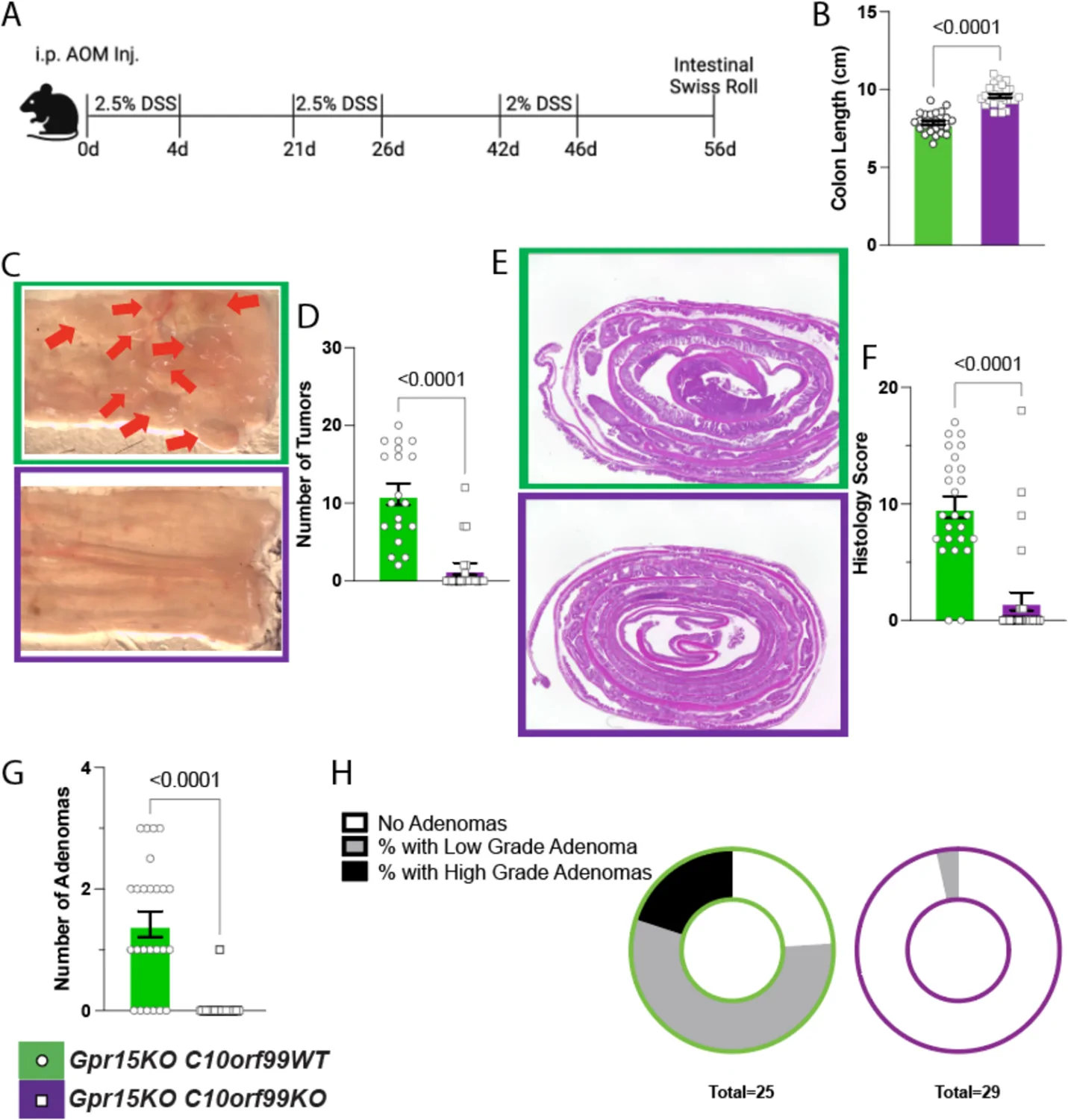
C10ORF99 exacerbates colitis-associated CRC independently of GPR15. **(A)** Schema of the experimental procedure administering AOM via i.p. injection as well as 3 cycles of DSS to cohoused SKO and DKO mice. **(B)** Average colon length from the end of the cecum to the rectum (n = 24–25). **(C)** Representative images and **(D)** tumor count of macroscopic tumors observed using a dissection microscope (n = 19–20). **(E)** Representative images and **(F)** histology scores taken from colon sections of SKO and DKO mice. (scoring criteria are in Table 2) (n = 25,29) **(G)** Number of observable adenomas from microscope slide sections of colon tissue (n = 25,29). **(H)** Histological grade of adenomas observed in SKO and DKO mice colon sections. Data are shown as mean ± SEM. Colon lengths were compared using Student’s *t*-test. Number of tumors, histology scores, and number of adenomas were compared using Mann-Whitney tests. Cohoused mice were used for these experiments.

In addition to the risk from colitis complications, IBD can significantly increase the risk of developing colitis-associated colorectal cancer.^3,7^ Since C10ORF99 deficiency protects mice from DSS-induced colitis (Fig. 2), we hypothesized that C10ORF99 deficiency could also protect mice from developing DSS colitis-induced colorectal cancer through GPR15-independent mechanisms. To test this, we utilized our SKO and DKO mice with an azoxymethane (AOM)-DSS model of colorectal cancer (Fig. 5A). As expected, the SKO had decreased colon length as compared to the DKO, indicating a greater level of inflammation after DSS treatment (Fig. 5B). We visualized tissue under a dissection microscope to determine the number of macroscopic tumors and found an increased number of tumors in SKO mice compared to DKO mice (Fig. 5C and 5D). Consistent with these results, we also found that the SKO mice have increased pathology in the colon compared to DKO mice from histology analysis (Fig. 5E and 5F) and that the SKO mice exhibited an increased number of adenomas and high-grade adenomas compared to DKO mice (Fig. 5G and H). These results indicate that, in addition to being protective during DSS-induced colitis, a C10ORF99 deficiency is also protective in the development of colitis-associated colorectal cancer.

## Discussion

In this study, we demonstrated that C10ORF99 is a non-redundant ligand of GPR15 in mediating T cell trafficking to the large intestine (Fig. 1 and S3). This frames the GPR15-C10ORF99 pathway as a potentially valuable target for future IBD treatments and provides insight into the maintenance of local homeostasis in the large intestine. However, the impact of targeting the GPR15-C10ORF99 pathway on patients with UC is still uncertain, given that GPR15 is expressed in both Tregs and T effector cells.^15,17^ Notably, the expression patterns of GPR15 differ between laboratory mice, which show a higher proportion of GPR15 + Tregs,^15^ and adult humans, who exhibit more GPR15 + T effectors.^15,17^ Contrary to earlier hypotheses^17^, the discrepancy is unlikely due to species-specific differences in the mechanisms regulating GPR15 expression, as it turned out that the aryl hydrocarbon receptor (AhR)-mediated control of GPR15 expression is conserved across species.^18,34,35^ Instead, the variation in GPR15 expression patterns likely results from differing environmental exposures that influence the immune system.^18,36,37^ Therefore, therapies targeting the GPR15-C10ORF99 pathway may yield variable effects in younger versus adult patients.

However, equally important to consider is the additional role of C10ORF99, which is independent of GPR15 functions. We uncovered that an *in vivo* GPR15-independent function of C10ORF99 exists in the large intestine. We found that C10ORF99 is produced by the epithelium (Fig. 4A) as well as the endothelium in the large intestine (Fig. 1). We discovered that C10ORF99 deficiency leads to protection from chemically induced colitis and that this function is independent of GPR15 (Fig. 2). This is likely because C10ORF99 inhibits the proliferation of intestinal epithelial stem cells (Fig. 3), resulting in a reduced ability of the colonic epithelium to recover from damage (Fig. 2). While C10ORF99 appears capable of inhibiting proliferation without requiring outside influence, microbiota may still be able to influence the effect size of C10ORF99′s influence, or mask C10ORF99′s effects altogether (Fig. S2D). It needs to be clarified whether C10ORF99′s effect on epithelial proliferation is the sole driver of the resistance to DSS colitis in *C10orf99* KO mice. For example, while we attribute the increased CD45^+^ cells in SKO compared to DKO mice to a result of poor epithelial recovery and thus more inflammation, it is possible that C10ORF99 influences inflammatory signaling, increasing inflammation through another mechanism.

A question that remains is the evolutionary benefit of C10ORF99, which slows the recovery of the colonic epithelium upon damage. It may be that C10ORF99 acts as a regulator of stem cell proliferation to retain stemness. There is evidence that the intestines contain a subpopulation of quiescent stem cells, which divide slower than the typical stem cells.^38^ It is possible that C10ORF99 influences the differentiation of the rapidly dividing LGR5-expressing stem cells to these more slowly dividing LGR5-expressing stem cells. With more rapidly dividing ISCs, the DKO may have increased protection from DSS-induced colitis. Future experiments should be conducted to look for signatures and populations of these quiescent stem cells in SKO vs. DKO mice to determine if there are differences in the presence of C10ORF99.

Alternatively, the colonic epithelium is one of the most highly proliferating cell groups in the body. Thus, it is reasonable to assume that this would require mechanisms to prevent any abnormalities from occurring during this frequent proliferation. While C10ORF99 deficiency increased adenoma formation in the inflammation-induced CRC model in our AOM/DSS experiments (Fig. 5), the evolutionary benefit of allowing C10ORF99 to inhibit stem cell proliferation may be to prevent the occurrence of neoplasia during the normal regeneration of epithelium without inflammation. While our findings in AOM/DSS model appear counterintuitive, they may depend on the inflammatory status of the cancer model being tested. Thus, to determine if C10ORF99 functions as a tumor suppressor for the large intestine epithelium, further studies need to be conducted using genetic colon tumor models rather than inflammation-based models, as shown here (Fig. 5). These studies would also require differentiation between tumor growth and initiation, as *C10orf99* appears to be downregulated in some colon tumor tissues.^22^ Therefore, C10ORF99 may be important in controlling tumor initiation, but may not be effective in controlling already formed tumor tissue.

All together, we propose here that the GPR15-independent roles of C10ORF99 should be taken into consideration when targeting the GPR15-C10ORF99 pathway for therapeutic purposes to treat UC. Our work demonstrates that C10ORF99 impedes the proliferation of intestinal stem cells in the large intestine; thus, any inhibition or alteration of C10ORF99 function would influence epithelial regeneration in the large intestine. C10ORF99 may also influence mast cells and neurons via Mas-related G protein-coupled receptors, as reported in the skin^24^, although our data suggest that mast cell recruitment is not influenced by the loss of C10ORF99 in the large intestine (Fig. S3). Given that adult humans have increased GPR15-expressing Th17 cells^15,17^, it seems likely that blockade of C10ORF99 would be beneficial in treating UC via GPR15-dependent and GPR15-independent mechanisms. However, the exact nature and the impact of C10ORF99 on the proliferation and differentiation of ISCs in the large intestine need further investigation.

## Methods

### Ethics

All animal studies were performed according to the protocol approved by the Institutional Animal Care and Usage Committee (IACUC) of Thomas Jefferson University.

### Mice

All mice used were on C57BL/6 background. Wild-type C57BL/6 mice were obtained from Jackson Laboratory. *Gpr15*^*gfp/gfp*^ mice were described previously.^15^ To generate *C10orf99* KO mice, we inserted premature STOP codon in the exon 1 of *C10orf99* gene by injecting CRISPR mRNA, two SgRNAs (5′-GAGCAGCATGCAGAGCagACCGG-3′ and 5′-CTGTTTCTGCATTTTCtcCTAGG-3′ and repair template (CGGCTCCTTCACTATGAGACTTCTAGCCCTTTCCGGTCTGCTCTGCATGCTGCTCCTCTGatagataagtgaCCATGGTTTCTGCATTTTCTCCTCA-GAAGGTAACCTGCCCCTGGGCATTGACTCCGTGGTCTGGGA) to zygotes from C57BL/6 mice in New York University transgenic core. For genotyping, the following primers were used for PCR reactions: 5′-TGCTGCTCCTCTGTTTCTGC-3′ and 5′-TCAATTCTTCTCATCCTGTCC CTC-3′ for wild-type allele (a product of 619 bps is expected); 5′-TGCTCCTCTGATAGATAAGTGACC-3′ and 5′-TCAATTCTTCTCATCC TGTCCCTC-3′ for KO allele (a product of 635 bps is expected) (Fig. S1). *Lgr5*^*eGFP-IRES-creERT2*^ mice were obtained from Jackson Laboratory (#008875) and crossed with *C10orf99*^*n/n*^ mice. For the analysis, mice were euthanized by CO2 gas or by exsanguination/perfusion according to the IACUC-approved animal protocol. Sex was not considered in this study design as preliminary data did not suggest differences in phenotype based on sex. We have generated SKO and DKO mice by crossing *C10orf99* Het and *Gpr15*^*gfp*^ Het mice. Once we obtained littermate founders of SKO and DKO mice, we intercrossed them to get enough SKO and DKO mice for performing experiments. To minimize the microbiota drift, we co-housed them during weaning until finishing the experiments. If littermate controls were used, it was specified in the figure legends.

### RNA sequencing

RNA sequencing of the sorted cells was performed in HudsonApha Institute for Biotechnology, Huntsville, AL 35806, USA. Data analysis was performed by GeneSpring software.

### Reporter assay for GPR15

PathHunter CHO-K1 cell line expressing GPR15-tagged with Pro-LinkTM and b-Arrestin tagged with enzyme acceptor was purchased from DiscoverX, Eurofins. Recombinant C10ORF99, chemerin, CCL11, CCL25 were purchased from R&D systems or Peprotech. Reporter assays for GPR15 were performed according to the manufacturer’s manual (DiscoverX, Eurofins).

### Transwell migration assay

Wehi-231 (CRL-1702) was purchased from ATCC and transduced with human GPR15 by retrovirus. 2 × 10^3^ cells were plated in the upper chamber of the transwell plate (Corning 3421 with 5 μm pores) in RPMI with 10 % serum. After 2 hrs of incubation at 37 °C, the plates were spun at 100x g to collect the cells in the bottom chamber and the cell numbers were counted.

### In vivo competitive homing assay

Short-term competitive homing assay was performed as described previously. CD4+ native T cells (CD4^+^CD62L^hi^CD44^lo^CD25^−^) were sorted from B6.SJL-PtprcaPep3b/BoyJ (The Jackson laboratory) or from C57BL/6J (The Jackson laboratory), and stimulated with 0.25 μg/ml of anti-CD3e (145–2C11, eBiosciences) and 1 μg/ml of anti-CD28 (37.51, eBiosciences) antibodies crosslinked by plate-bound anti-hamster IgG (MPBiochemical). These cells were transduced from day 1 and day2 after stimulation with GPR15-expressing retrovirus or empty retrovirus, respectively. The viral backbone has IRES-THY1.1 and therefore transduced cells could be tracked with THY1.1 staining. At day 3, cells were washed and plated in the presence of 100 U/ml of IL-2 (eBiosciences). At day 5, live cells were enriched by lymphocyte-M (Cedarlane). The congenic cells were mixed at 1:1 ratio and 20–30 million mixed cells were transferred to C57BL/6J recipient mice intravenously. All donors express THY1.1 while GPR15-expressing donors have CD45.1 and control-expressing donors have CD45.2. Donor cells were analyzed within 12–18 hrs after the adoptive transfer.

### DSS-induced colitis

Dextran sulfate sodium (#DB001, TdB labs) was used to induce colitis. Mice were subjected to 10 days of 1.5 % DSS for males and 2 % DSS for females in drinking water with 15 g/L of grape-flavored Kool-Aid. At Day 10, DSS water was removed, and normal water was provided. Mice were analyzed at the peak of inflammation (day 14) for colon length measurements and preparation of intestinal Swiss Rolls (Fig. 2).

### TNBS-induced colitis

Mice were anesthetized through i.p. injection of ketamine/xylazine. The area on the back of the skin, between the shoulders, was shaved. 2 % of 2,4,6-trinitrobenzene sulfonic acid (Fisher 18–605–217) in a mix of acetone and olive oil (4:1) was applied to the skin for sensitization. At 14-days after sensitization, mice were fasted for 12–16 h, and 2 % TNBS was administered through intra-rectal injection. Mice were analyzed 7 days after re-challenge.

### H&E staining, histopathological analysis

Representative sections with Hematoxylin/Eosin staining were evaluated blindly by a gastrointestinal pathologist for the following criteria in Table 1. Pathology score was calculated by combining the scores from readings on neutrophil infiltration, edema, goblet cell depletion, crypt damage, atrophy/crypt loss, epithelial regeneration, and erosions/ulceration, as described previously.^18^

### Cell preparation from the intestines

Large intestine lamina propria immune cells were prepared as previously described^18^. Cells were analyzed using a BD Fortessa or BD Symphony.

### FITC-dextran assay for intestinal permeability

Mice were assayed as previously described^39^.

### Measurement of intestinal crypt lengths

Intestinal segments were divided into 3 segments (proximal, mid, and distal). For each segment, 3 regions were observed. In each region, 30 crypt lengths were measured, yielding 90 measurements/segment and 270 measurements/mouse. A mean crypt length was calculated for each segment as well as for the total bowel. In Fig. 3A, data are shown with each data point representing an individual mouse crypt length average; however, in Fig. S2E, data are shown with each data point representing an individual crypt length. Slides were observed with a BZ-X series fluorescence microscope and analyzed using BZ-X Viewer.

### EdU administration and staining

For quantifying EdU incorporated cells (Fig. 3C) Mice were injected intraperitoneally with 100 μL of 2 mg/mL EdU. After 24 h, mice were euthanized through exsanguination and perfusion. For measuring distance traveled by EdU incorporated cells, mice were injected with 100 μL of 20 mg/mL EdU (Fig. 3D). Mice were euthanized after 48 h. Tissues were fixed overnight in 4 % PFA at 4 °C and then were washed twice in PBS. Fixed tissues were dehydrated in 70 % ethanol. Tissues were then embedded in paraffin, 4–6 μm sections were cut 400 μm apart by the Thomas Jefferson University Translational Research/Pathology Shared Resource. Staining for EdU was conducted using the Click-iT EdU Alexa Fluor 594 Imaging Kit (ThermoFisher Scientific, C10339). Images taken using a BZ-X series fluorescence microscope were analyzed using ImageJ software.

### TUNEL staining

Intestinal Swiss rolls were prepared according to the same procedure as described for H&E staining and pathological analysis. These were embedded in paraffin, sectioned, and TUNEL assays were performed by the Thomas Jefferson University Translational Research/Pathology Shared Resource with the Click-iT TUNEL Colorimetric IHC Detection Kit (ThermoFisher Scientific, C10625).

### Intestinal epithelial organoid cultures

Organoid cultures were established as previously described.^33,40^ LGR5-GFP^+^ cells were sorted using a BD FACS ARIA. Organoids were cultivated in L-WRN media, which has been described previously.^41^ Recombinant C10ORF99 was obtained from Peprotech (300–72) and added to L-WRN media at various concentrations. At 6 days, organoid pictures were taken under a dissection microscope, and diameter measurements were taken using ImageJ software.

### MTT procedure

5000 ATCC SW480 cells were plated per well onto 96-well plates. After 24 h, full media was swapped for ½ serum media with rC10ORF99 added or ½ serum media untreated controls. An initial 0 d MTT was conducted followed by another MTT assay every 24 h for 6 days. MTT’s were conducted according to the manufacturer’s protocol (Sigma Aldrich #11465007001).

### Wound healing assay procedure

ATCC CMT93 mouse rectal tumor cells were plated on 12-well plates and grown overnight to confluency. Then, using a 200 μL pipette tip, a wound was cut into the culture, and the percentage of wound closure was observed after 4 h of growth. The wound closure percentage was calculated by ((initial wound width – wound width after 4 h) / initial wound width) x100 = Percentage of wound closure. Cells were left untreated or treated with 100 nM mouse recombinant C10ORF99 (Peprotech #300–72).

### Antibiotic treatment of mice

For antibiotic treatment, a mixture of ampicillin (1 g/L), vancomycin (0.5 g/L), metronidazole (1 g/L), neomycin (1 g/L), and Kool-Aid (Grape flavor, 20 g/L) was filtered through 0.22 μm and provided in the drinking water.

### Tumor induction

AOM-DSS Colitis-induced cancer was induced as previously described^42^. Mice were analyzed at 56-days post AOM injection. Tissues were fixed, embedded in paraffin, mounted on slides, and stained with H&E. Tumor counts and measurements were performed in a blinded fashion. Measurements were conducted using ImageJ software. Additionally, slides were analyzed by a pathologist in a blinded fashion for criteria specified in Table 2.

#### Antibodies used:

FITC-CD26 (1:100 dilution; Clone H194–112, Biolegend 137805), PerCPCy5.5-CD3ε (1:300 dilution; Clone 500A2 Biolegend 152302), PerCPCy5.5-Thy1.2 (1:1000 dilution; Clone 30-H12, Biolegend 105337), PerCPCy5.5-CD19 (1:200 dilution; Clone 1D3, Biolegend 152405), PerCPCy5.5-B220 (1:300 Dilution; Clone RA3–6B2, Biolegend 103235), PerCPCy5.5-NK1.1(1:300; Clone PK136, Biolegend 108725), PE-CD172 (1:200 Dilution, Clone P84, Biolegend 144011), PE-Cy-7-FceR1 (1:400 Dilution; Clone MAR-1, Biolegend 134317), APC-CD64 (1:200 Dilution; Clone X54–5/7.1, Biolegend 139305), Alexa-Fluor700-I-A/I-E (1:1250 Dilution; Clone M5/114.15.2, Biolegend 107621), Alexa750-Ly6c (1:400 Dilution; Clone HK1.4, Biolegend 128045), Pacific Blue-Ly6G (1:1000 Dilution; Clone 1A8, Biolegend 127612), Brilliant Violet 510-CD11c (1:100 Dilution; Clone N418, Biolegend 117337), Brilliant Violet 650-CD45 (1:4000 Dilution; Clone 30-F11, Biolegend 103151), Brilliant Violet 711-CD11b (1:200 Dilution; Clone M1/70, Biolegend 101241), Brilliant Violet 785-c-Kit (1:400 Dilution; Clone ACK2, Biolegend 135138), Biotinylated-SiglecF (1:1000 Dilution; Clone S17007L, Biolegend 155512), FITC-KLRG-1 (1:1000 Dilution; Clone 2F1/KGLR1, Biolegend 138409), PerCPCy5.5-CD172 (1:200 Dilution; Clone P84, Biolegend 14409), PE-FoxP3 (1:1:200 Dilution; Clone FJK-16 s, Thermofisher 12–5773–82), PE-CD8b (1:400 Dilution; Clone YTS156.7.7, Biolegend 126607), PE-Cy7-TCRgd (1:400 Dilution; Clone GL3, Biolegend 118123), APC-RORgt (1:200 Dilution; Clone AFKJS-9, Thermofisher 17–6988–82), APC/Fire750-TCRb (1:400 Dilution; Clone H57–597, Biolegend 109246), Pacific Blue-Thy1.2 (1:1000 Dilution; Clone 53–2.1, Biolegend 140306), Brilliant Violet 510-CD4 (1:400 Dilution; Clone RM4–5, Biolegend 100553), Brilliant Violet 605-NK1.1 (1:400 Dilution; Clone PK136, Biolegend 108739) Brilliant Violet 711-Sca-1 (1:100 Dilution; Clone D7, Biolegend 108131), Brilliant Violet 785-NKp46 (1:200 Dilution; Clone 29A1.4, Biolegend 137637), Biotinylated-F4/80 (1:1000 Dilution; Clone BM8, Biolegend 123105), Biotinylated-CD19 (1:1000 Dilution; Clone 1D3, Biolegend 152420), Biotinylated-CD11c (1:1000 Dilution; Clone N418, Biolegend 117303), Biotinylated-FceR1 (1:1000 Dilution; Clone MAR-1, Biolegend 134303), Biotinylated-Ly6c/G (1:1000 Dilution; Clone RB6–8C5, Biolegend 108403), PerCPCy5.5-SiglecF (1:1000 Dilution; Clone S17007L, Biolegend 155512), Brilliant Violet 605-CD19 (1:400 Dilution; Clone 6D5, Biolegend 115539), Biotinylated-CD3e (1:300 Dilution; Clone 145–2C11, Biolegend 100303), Biotinylated-NK1.1 (1:300 Dilution; Clone PK136, Biolegend 108703), Biotinylated-Thy1.2 (1:1000 Dilution; Clone 30-H12, Biolegend 105304), PerCPCy5.5-CD69 (1:200 Dilution; Clone H1.2F3, Biolegend 104521), PE-Cy7-EpCAM (1:4000 Dilution; Clone G8.8, Biolegend 118215).

### Statistical analysis

The normality of data distribution was examined using the Kolmogorov-Smirnov test or the Shapiro-Wilk test to determine which statistical comparisons to use: T-tests or Mann-Whitney tests for comparisons with two populations, and one-way ANOVA for groups with more than two populations, followed by Tukey’s multiple comparisons test. Graphs were created using GraphPad Prism (v10).

## Supplementary Material

Supplementary Material

## Figures and Tables

**Table 1:** Criteria for evaluating DSS pathology (Fig. 2).

Score	0	1	2	3
**Neutrophil infiltration**	None	1–50	50–100	100-
**Edema**	None	mild	moderate	Severe
**Goblet cell depletion**	>50/HPF	25–50/HPF	10–25/HPF	<10/HPF
**Crypt damage**	intact	basal1/3	basal 2/3	entire loss
**atrophy/crypt loss**	normal crypt	mild	moderate	severe
**Epithelial regeneration**	Complete	Slight injury	Surface not intact	No tissue repair
**Epithelial hyperplasia**	None	1–50 %	51–100 %	>100 %
**Erosions/ulceration**	None	Focal, lamina propria	Muscularis propria	Full thickness

**Table 2:** Criteria for evaluating AOM-DSS pathology (Fig. 5).

Score	0	1	2	3
**Neutrophil infiltration**	None	1–50	50–100	100-
**Edema**	None	mild	moderate	Severe
**Goblet cell depletion**	>50/HPF	25–50/HPF	10–25/HPF	<10/HPF
**Crypt damage**	intact	basal1/3	basal 2/3	entire loss
**atrophy/crypt loss**	normal crypt	mild	moderate	severe
**Epithelial regeneration**	Complete	Slight injury	Surface not intact	No tissue repair
**Epithelial hyperplasia**	None	1–50 %	51–100 %	>100 %
**Erosions/ulceration**	None	Focal, lamina propria	Muscularis propria	Full thickness
**No dysplasia**	Yes/No			
**Low-grade dysplasia**	Yes/No			
**High-grade dysplasia**	Yes/No			
**Adenocarcinomas**	Yes/No			

## Appendix A. Supplementary data

Supplementary data to this article can be found online at https://doi.org/10.1016/j.mucimm.2025.08.001.
