# Supplementary material for: C10ORF99 (GPR15L) increases susceptibility to colitis and colitis-induced colorectal cancer via GPR15-independent mechanisms, while mediating GPR15-dependent T cell migration to the large intestine
Source: Mucosal Immunol. Author manuscript; Available in PMC 2026 Sep 10. (PMC13559717; doi:10.1016/j.mucimm.2025.08.001)
Supplement: Supplementary file 1 [file NIHMS2208306-supplement-Supplementary_Material.docx]

**Supplemental Fig. 1. Development and validation of *C10orf99KO* mice. (A)** Representative flow plots of sorting strategy to isolate the large intestine endothelial cells (LIEN), the small intestine endothelial cells (SIEN), and the high endothelial venule (HEV) cells in the lymph nodes for the comparison of expression profiles*.* **(B)** Strategy of creation of *C10orf99* KO mice. A premature STOP codon was inserted into exon 1 of the *C10orf99* gene. **(C)** Validation of the *C10orf99KO* mice showing that loss of C10ORF99 impairs GPR15-mediated trafficking of Tregs to the large intestine (n=5, with littermate controls). Data are shown as mean ± SEM. Populations were compared using Student’s t-test.

**Supplemental Fig. 2. DSS-induced colitis with *Gpr15* WT mice and TNBS-induced colitis in SKO vs DKO. The effect of recombinant C10ORF99 on colon epithelial cell growth, and the effect of antibiotics on the crypt lengths of SKO vs DKO. (A)** DSS was administered in drinking water to induce colitis in cohoused *C10orf99* WT *and C10orf99* KO mice on a *Gpr15* WT background (n=7-8). Weight change was recorded over 14 days, with 10 days of DSS drinking water and 4 days of regular water. **(B)** SKO and DKO cohoused mice were subjected to a TNBS hypersensitivity model of colitis (n=13). Mice were sensitized with 2% TNBS on the skin. After 2 weeks, the mice were re-challenged, and weight change was recorded. **(C)** MTT assay of SW480 cells without treatment or with human 100 nM rC10ORF99. **(D)** Wound healing assay of CMT93 mouse tumor cells without treatment or with 100 nM mouse rC10ORF99, showing the percentage of wound closure after 4 hours. **(E)** Quantitative analysis of large intestine crypt lengths of cohoused SKO and DKO mice with antibiotic-treated water or untreated, plotted as individual crypt lengths rather than averages by mouse as in Fig. 3A (n=1080 crypts for total, n=360 crypts for each segment). Populations were compared using Student’s t-test. Cohoused mice were used for these experiments.

**Supplemental Fig. 3 SKO and DKO are largely similar in immune cell populations in the large intestine. (A)** Representative flow plots for the identification of immune cell populations between cohoused SKO and DKO mice. **(B)** Comparison of immune cell populations between SKO and DKO mice at steady state (n=4-5). **(C)** Comparison of immune cell populations between SKO and DKO mice following administration of DSS (n=4-5). Data are shown as mean ± SEM. Populations were compared using Student’s t-test. Cohoused mice were used for these experiments.
